# Somatoform Dissociative Symptoms Have No Impact on the Outcome of Trauma-Focused Treatment for Severe PTSD

**DOI:** 10.3390/jcm10081553

**Published:** 2021-04-07

**Authors:** Harmen A. Zoet, Ad de Jongh, Agnes van Minnen

**Affiliations:** 1 PSYTREC, 3723 MB Bilthoven, The Netherlands; a.d.jongh@acta.nl (A.d.J.); vanminnen@psytrec.com (A.v.M.); 2 Academic Centre for Dentistry Amsterdam (ACTA), University of Amsterdam and VU University Amsterdam, 1081 LA Amsterdam, The Netherlands; 3 School of Health and Society, University of Salford, Salford M6 6PU, UK; 4 Institute of Health and Society, University of Worcester, Worcester WR2 6AJ, UK; 5 School of Psychology, Queen’s University, Belfast BT7 1NN, UK; 6 Behavioural Science Institute (BSI), Radboud University, 6525 GD Nijmegen, The Netherlands

**Keywords:** posttraumatic stress disorder, somatoform dissociative symptoms, dissociation, intensive trauma-focused treatment, EMDR, prolonged exposure, compartmentalization

## Abstract

For patients with complex or other severe forms of PTSD, particularly in cases with dissociative symptoms, different treatment approaches have been suggested. However, the influence of somatoform dissociation on the effectiveness of trauma-focused treatment has hardly ever been studied. This study aims to test the hypotheses that (1) PTSD patients reporting a low level and those reporting a high level of somatoform dissociative symptoms would both benefit from an intensive trauma-focused treatment, and that (2) somatoform dissociative symptoms would alleviate. Participants were 220 patients with severe PTSD, enrolled in an intensive treatment program combining EMDR therapy and prolonged exposure therapy, without a preceding stabilization phase. Trauma history was diversified, and comorbidity was high. PTSD symptoms (CAPS-5 and PCL-5) and somatoform dissociative symptoms (SDQ-5 and SDQ-20) were assessed at pre-treatment, post-treatment and at six months after completion of treatment. The course of both PTSD and somatoform dissociative symptoms was compared for individuals reporting low and for those reporting high levels of somatoform dissociative symptoms. Large effect sizes were observed regarding PTSD symptoms reduction for patients with both low and high levels of somatoform dissociation. Somatoform dissociation did not impact improvement in terms of PTSD symptom reduction. The severity of somatoform dissociative symptoms decreased significantly in both groups. This decrease was greater for those with a positive screen for a dissociative disorder. These results add further support to the notion that the presence of strong somatoform dissociative symptoms in patients with PTSD does not necessarily call for a different treatment approach. Clinical implications are discussed.

## 1. Introduction

For patients with severe or complex forms of PTSD, such as those with dissociative symptoms, it has been suggested that a modified treatment approach, i.e., a phase-based approach, might be more appropriate than regular trauma-focused treatment [1,2]. Treatment would then, for example, start with a stabilization phase focused on skill training in emotion regulation, working on competences in social interactions, and grounding skills in order to decrease dissociative symptoms, before commencing the trauma-focused part of the treatment (see, e.g., [3]). In this view, dissociative symptoms are considered a distinct form of PTSD. Indeed, in some studies it was found that PTSD patients with additional dissociative symptoms benefit less from a trauma-focused treatment than those without dissociative symptoms [4]. On the other hand, other studies found that dissociative symptoms did not predict trauma-focused treatment efficacy [5,6]. A recent meta-analysis showed that dissociation had no significant effect on treatment outcome, and that the impact of dissociation was about zero [7]. Additionally, some studies found that direct trauma-focused therapy actually led to a decrease in dissociative symptoms, e.g., [8,9]. Another view is, therefore, that dissociative symptoms are associated with PTSD, rather than being a distinct form of PTSD. Consecutively, it has been asserted that trauma-focused therapy should be considered as the first-choice treatment for PTSD patients with additional dissociative symptoms [5,10].

However, dissociation is a broad concept, referring to a variety of phenomena. In most studies hitherto, mainly psychological dissociation has been studied. Van der Hart, Nijenhuis, Steele, and Brown [11] pointed out that besides disruptions to mental functions and a subjective experience of an altered state of consciousness (psychological dissociation, e.g., derealization and depersonalization), disruptions in bodily functions and sensations, such as loss of motor control, analgesia, seizures, and pain, could also be considered forms of dissociation, referred to as somatoform dissociation. To clarify the use of the term dissociation, Holmes et al. [12] classified dissociative phenomena into two different qualitative categories: detachment and compartmentalization. The first category refers to an altered state of consciousness, i.e., psychological dissociative phenomena, whereas the latter refers to a separation of normally integrated mental processes, leading to a sudden inability to control certain actions or cognitive processes, i.e., somatoform dissociation [12,13]. Neurologically, detachment seems to be characterized by a top-down inhibition of limbic emotional systems and an increased activation of right prefrontal cortex regions, illustrative of a decrease in feelings of anxiety [12,14,15]. Compartmentalization, on the other hand, has been found to be related to reduced thickness of the left caudal anterior cingulate cortex [16], allegedly related to emotion regulation problems and fear extinction deficits [17,18], both important underlying processes for trauma-related treatment [19,20]. Compartmentalization might, therefore, reduce the likelihood of benefiting from trauma-focused treatment.

As with psychological dissociation, a phase-based treatment approach has been recommended for those with somatoform dissociative symptoms [21]. Thus far, however, the influence of somatoform dissociation on trauma-focused treatment outcomes in PTSD patients has hardly ever been studied. A study by Myers et al. [22] showed that prolonged exposure therapy for patients with PTSD and psychogenic non-epileptic seizures (PNES), a specific form of somatoform dissociation, was effective for both PTSD and PNES symptoms. Thus, although it is largely unknown how somatoform dissociation affects trauma-focused treatment outcomes in PTSD patients, some preliminary evidence suggests that somatoform dissociation, like psychological dissociation, is responsive to trauma-focused treatments.

The purpose of the present study was to determine the extent to which PTSD patients with somatoform dissociative symptoms would respond to trauma-focused treatment in comparison to patients without somatoform dissociative symptoms. Based on the above mentioned findings regarding conversion disorder and our previous studies with patients with psychological dissociation [9,23], we hypothesized that both patients reporting a low level and those reporting a high level of somatoform dissociative symptoms (i.e., fulfilling the criteria of a positive screen for a dissociative disorder) would benefit from an intensive trauma-focused treatment, and that the severity of somatoform dissociative symptoms would decrease significantly during treatment.

## 2. Materials and Methods

### 2.1. Participants

**Figure 1:**
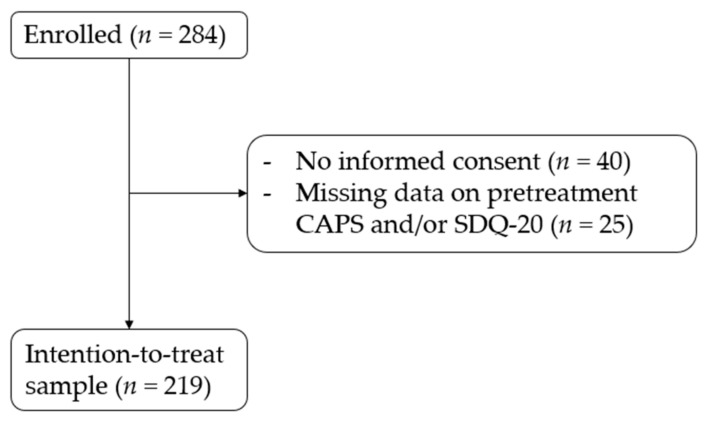
Flowchart for the intention-to-treat sample used for main analysis.

Participants were patients who were referred to an intensive treatment program at the Psychotrauma Expertise Centrum (PSYTREC) in the Netherlands. A total of 284 patients enrolled for treatment, of whom 244 gave informed consent for participation in our clinical research. Pre-treatment scores on the main measurements were missing for 25 patients, who were therefore excluded from the analyses. This resulted in a final intention-to-treat (ITT) sample of 219 patients (see also Figure 1).

### 2.2. Procedure

Pre-treatment assessment took place during intake using the Clinician-Administered PTSD Scale (CAPS), the Mini-International Neuropsychiatric Interview (MINI), and the Somatoform Dissociation Questionnaire-20 (SDQ-20). Additionally, the inclusion criteria were checked and when met, patients were asked to sign a treatment contract and informed consent form for research purposes. Inclusion criteria were being at least 18 years old, and having a PTSD diagnosis according to the 5th edition of the Diagnostic and Statistical Manual of Mental Disorders (DSM-5) [24]. Exclusion criteria were not having sufficient knowledge of the Dutch language to be able to complete the assessments, and a suicide attempt in the three months prior to intake. After the intake sessions, the intensive eight-day treatment program started. Daily assessment with the Somatoform Dissociation Questionnaire-5 (SDQ-5) and the Dutch version of the 20-item PTSD Checklist for DSM-5 (PCL-5) took place during the therapy session in the morning. Nine days after treatment, post-treatment assessments were carried out using both the CAPS and the SDQ-20. Assessors of all measures were blind to the study hypotheses. The study was performed in accordance with the principles of the Declaration of Helsinki. The Medical Ethics Review Committee of VU University Medical Centre (registered with the US Office for Human Research Protections (OHRP) as IRB00002991, FWA number FWA00017598) has confirmed that the Medical Research Involving Human Subjects Act (WMO) does not apply to this study. For this reason, an official approval of this study by the committee was not required.

### 2.3. Treatment

Treatment consisted of eight days and was provided over two weeks, with four treatment days per week. Patients received one Prolonged Exposure (PE) session of 90 min in the morning and one Eye Movement Desensitization and Reprocessing (EMDR) therapy session of 90 min in the afternoon. Therapy sessions were provided by psychologists who were trained in both forms of psychotherapy. EMDR therapy was provided according to the Dutch version of the EMDR standard protocol [25]. PE therapy was provided largely according to the protocol by Foa, Hembree, and Rothbaum [26], albeit the recording of the sessions and homework assignments were omitted due to the intensive treatment format. Importantly, no interventions were specifically aimed at reducing or coping with dissociative symptoms. Every patient was treated by multiple therapists, adhering to the principle of therapist rotation [27]. Additionally, patients participated during the day in physical activities in a group, and attended daily psychoeducation meetings in a group format. See Woudenberg et al. [28] for a detailed description of the treatment program.

### 2.4. Measurements

PTSD symptom severity was measured using the Dutch version of the Clinician-Administered PTSD Scale for DSM-5 (CAPS-5), a well-validated semi-structured diagnostic interview [29,30]. This consists of 30 items, corresponding to the DSM-5 PTSD symptoms, measured over the past month. The Somatoform Dissociation Questionnaire-20 (SDQ-20) [31] was used to measure the severity of somatoform dissociative symptoms. This 20-item questionnaire has been well-validated, and scoring has been proven robust to suggestion [32]. To operationalize high levels of somatoform dissociation, we used a cut-off score of 35, which was used in earlier studies as an indication of the possible presence of a dissociative disorder (positive screen group) [21,32]. Additionally, the SDQ-5 was administered on a daily basis during treatment. This 5-item questionnaire is derived from the SDQ-20 and has high sensitivity and specificity in the screening of somatoform dissociative symptoms [32]. The course of PTSD symptoms during treatment was measured using the Dutch version of the 20-item PTSD Checklist for DSM-5 over the past 24 h (PCL-5) [33,34], which has excellent reliability and validity [35]. Comorbidity was assessed using the Dutch version of the Mini-International Neuropsychiatric Interview (MINI) [36,37]. The MINI is a validated structured interview to classify DSM-IV disorders and assess suicidal risk [37,38].

### 2.5. Data Analyses

Analyses were performed using IBM SPSS Statistics for Windows (version 24). All analyses were carried out on the basis of intention-to-treat. To test the main hypotheses, we conducted a simple linear regression analysis with SDQ-20 scores at intake as the independent variable, and the difference between pre-treatment and post-treatment CAPS scores as the dependent variable. Additionally, a factorial mixed ANOVA was conducted, with the CAPS-5 and SDQ-20 scores, respectively, over time (pre-treatment, post-treatment) as the within-subjects factor, and a positive screen for a dissociative disorder according to the SDQ-20 (≥35 vs. <35) as the between-subjects factor. To analyze the course of symptoms during treatment, a mixed ANOVA was conducted with the daily PCL-5 scores and SDQ-5 scores, respectively, as the within-subjects factor, and the possible presence of a dissociative disorder, as suggested by the SDQ-20 scores, as the between-subjects factor. Because the assumption of sphericity was violated for the total PCL-5 scores (*χ*^2^ = 315.05, *p* < 0.001) and the total SDQ-5 scores (*χ*^2^ = 710.00, *p* < 0.001), Greenhouse–Geisser corrections were applied. Cohen’s [39] rule of thumb was used to interpret Cohen’s *d* effect sizes. Because only limited follow-up data were available at 6 months follow-up (*n* = 119), we explored the influence of somatoform dissociative symptoms on treatment outcome at 6 months follow-up in a separate analysis.

## 3. Results

### 3.1. Patient Characteristics

At pre-treatment, 13.2% of the patients scored ≥ 35 on the SDQ-20, indicative of a dissociative disorder. Patients scoring above and below this cut-off score on the SDQ-20 at intake did not differ in age, *t*(217) = 1.60, *p* = 0.11, or gender, *χ*^2^(1) = 0.93, *p* = 0.33, nor was there a difference in the proportion that experienced physical abuse, *χ*^2^ (1) = 1.47, *p* = 0.23, or armed violence, *χ*^2^(1) = 0.58, *p* = 0.45. Patients in the positive screen group had experienced sexual abuse more often, *χ*^2^ (1) = 7.06, *p* = 0.01, and 75.9% also met the criteria for the dissociative subtype of PTSD, which was significantly more than those in the negative screen group (41.4%), *χ*^2^ (1) = 11.90, *p* = 0.001. A total of 14 patients (6.4%) dropped out of treatment; this proportion was not different between the two subgroups, *χ*^2^(1) = 0.48, *p* = 0.49. Sample characteristics can be found in Table 1.

### 3.2. Influence of Somatoform Dissociation on Treatment Outcome

**Figure 2:**
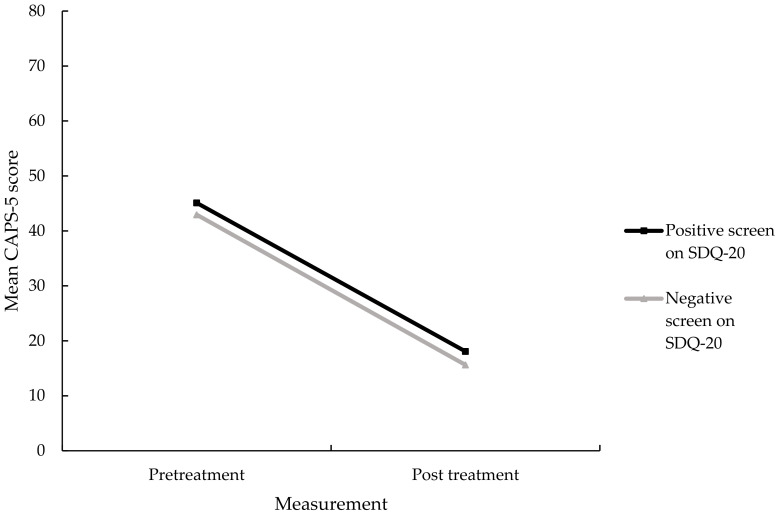
Mean CAPS-5 scores for those with and those without a positive screen on the SDQ-20, *n* = 219. CAPS-5, Clinician-Administered PTSD Scale for DSM-5.

A simple linear regression, using pre-treatment SDQ-20 scores as the independent variable, and the difference between pre-treatment and post-treatment CAPS scores as the dependent variable, showed that the severity of somatoform dissociative symptoms was not associated with treatment outcome, *F*(1, 211) = 0.15, *p* = 0.67, R^2^ = 0.001. A significant main effect of time on decreases in PTSD symptom severity (i.e., CAPS-5 scores) was found, *F*(1, 217) = 323.03, *p* < 0.001, Cohen’s *d* = 2.34. No significant main effect of group was found, *F*(1, 217) = 1.72, *p* = 0.19, which indicates that there was no significant difference between both groups in terms of PTSD symptom decrease. Additionally, no significant interaction effect was found between the groups and total CAPS-5 scores, *F*(1, 217) = 0.09, *p* = 0.93, suggesting that a pre-treatment positive screen for a dissociative disorder did not affect treatment outcomes in terms of a decrease in PTSD symptoms (see Figure 2). Means and SDs on both points of measurement can be found in Table 2.

**Figure 3:**
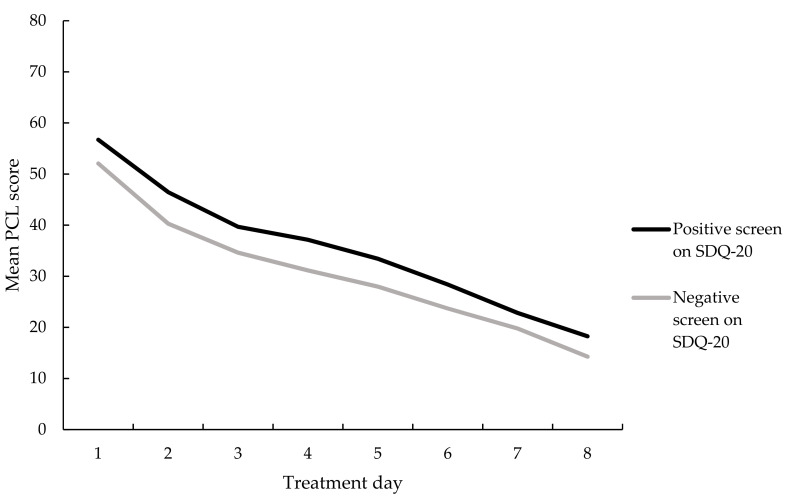
Mean PTSD Checklist for DSM-5 (PCL-5 scores) per treatment day for those with and without a positive screen on the SDQ-20, *n* = 201.

Regarding PCL-5 scores during treatment, a significant main effect of time was found, i.e., total PCL-5 scores decreased significantly during treatment, *F*(4.42, 879.44) = 160.78, *p* < 0.001, partial η^2^ = 0.45. No significant main effect of group was found, suggesting no significant differences in PTSD symptom reduction between those in the positive screen group and those in the negative screen group, *F*(1, 199) = 3.74, *p* = 0.055, see Figure 3. No significant interaction effect was found between the presence of a positive screen for a dissociative disorder according to the SDQ-20 and daily PCL-5 scores, *F*(4.42, 879.44) = 0.29, *p* = 0.90.

### 3.3. Change in Somatoform Dissociative Symptoms Associated with Treatment

**Figure 4:**
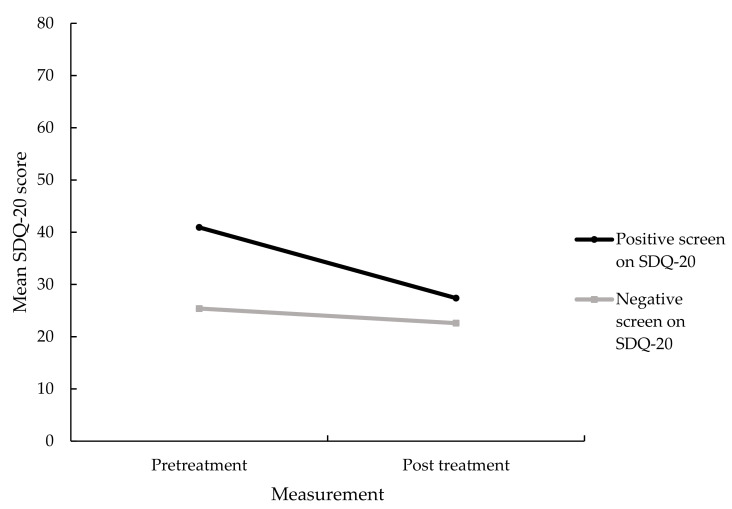
Mean SDQ-20 scores for those with and without a positive screen on the SDQ-20, *n* = 219.

A significant main effect of time on the severity of somatoform dissociative symptoms was found, *F*(1, 217) = 260.33, *p* < 0.001, Cohen’s *d* = 0.73. Additionally, a significant interaction effect was found between a positive screen for a dissociative disorder according to the SDQ-20 and total SDQ-20 scores, *F*(1, 217) = 112.55, *p* < 0.001, partial η^2^ = 0.34, suggesting that patients of the positive screen group showed a significantly greater decrease in somatoform dissociative symptoms after treatment, as shown in Figure 4. Using the same cut-off score as at intake, six patients (20.7%) still fulfilled the criteria of a positive screen for a dissociative disorder based upon the SDQ-20 at post-treatment. A McNemar test showed that this was a significantly smaller proportion of patients than before treatment, *χ*^2^ = 17.93, *p* < 0.001. Furthermore, a significant positive correlation was found between the decrease in PTSD symptoms and the decrease in somatoform dissociative symptoms, *r*(212) = 0.19, *p* = 0.005. See also Table 2 for mean SDQ-20 scores.

**Figure 5:**
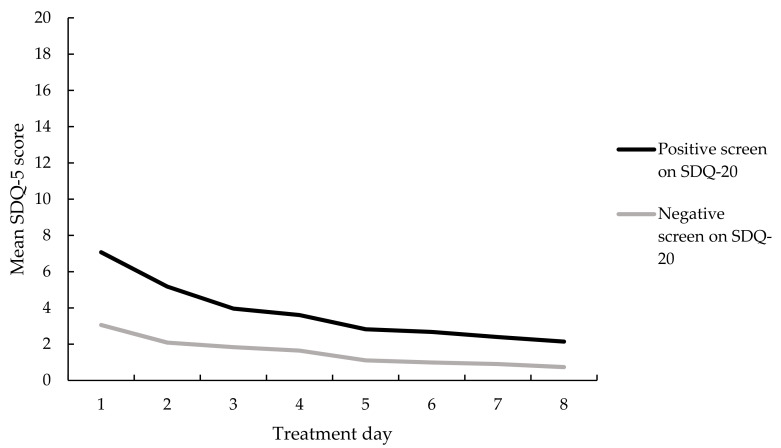
Mean SDQ-5 scores per treatment day for those with and those without a positive screen on the SDQ-20, *n* = 199.

SDQ-5 scores decreased significantly over the treatment days, *F*(3.07, 603.82) = 53.97, *p* < 0.001, partial *η*^2^ = 0.22. Additionally, a significant main effect of group was found, *F*(1, 197) = 21.90, *p* <0.001, partial η^2^ = 0.10, indicating that those in the positive screen group had higher SDQ-5 scores on all days than those in the negative screen group. Moreover, an interaction effect was found, *F*(3.07, 603.82) = 7.45, *p* < 0.001, partial η^2^ = 0.04, indicating a significantly larger decrease in somatoform dissociative symptoms for those with a positive screen compared to those with a negative screen (see Figure 5).

### 3.4. Exploration of Follow-Up Data

To further investigate the potential influence of pre-treatment somatoform dissociative symptoms on PTSD symptoms at 6 months’ follow-up, we conducted a simple regression analysis with total pre-treatment SDQ-20 scores as the independent variable, and the difference in total CAPS scores between pre-treatment and follow-up as the dependent variable. No significant relationship was found, *F*(1, 117) = 3.69, *p* = 0.06, R^2^ = 0.03.

## 4. Discussion

The first aim of the present study was to determine the extent to which patients reporting a high level of somatoform dissociative symptoms, compared with those reporting a low level, would benefit from an intensive trauma-focused treatment. The results showed that PTSD symptom severity decreased significantly in both groups, with no differences in PTSD symptom decrease between the groups. In addition to the pre- and post-treatment results, during the treatment days the decrease in self-reported PTSD symptoms also did not differ between the two groups. Furthermore, the severity of somatoform dissociation at pre-treatment was not predictive of treatment outcome at post-treatment and six-months follow-up in terms of PTSD symptom reduction.

These findings are in line with studies on psychological dissociative symptoms (see [7]), in that dissociative symptoms do not seem to have a significant impact on the decrease in PTSD symptoms when trauma-focused therapy is administered without treating dissociative symptoms first. To our knowledge, this is the first study that extends these meta-analytic findings to somatoform dissociation. Both the lack of difference in PTSD symptom reduction between the two groups and the fact that the low drop-out rate did not differ between the two groups suggest that trauma-focused therapy is well-tolerated by this subgroup of PTSD patients, and proved not, as some say, too overwhelming [21,40,41]. Emotional overmodulation, and the expected co-occurring difficulties with fear activation, might be less present among those with somatoform dissociative symptoms than assumed [14,15]. This would imply that it is not imperative in treatment to first address the somatoform dissociative symptoms before commencing with trauma-focused therapy, because it does not notably impede trauma-focused therapy. Hence, when replicated, the results of our study indicate that trauma-focused therapy can also be applied as a first-choice treatment for PTSD patients with additional somatoform dissociative symptoms.

The second aim of this study was to determine the extent to which somatoform dissociative symptoms would decrease during treatment. The results showed that somatoform dissociative symptoms decreased significantly, and that this decrease was larger for those with high levels of somatoform dissociative symptoms. During the treatment program, the decrease in somatoform dissociative symptoms was higher for those with high levels of somatoform dissociation at pre-treatment. Interestingly, 23 (79.3%) of the 29 patients who had high levels of somatoform dissociative symptoms at pre-treatment no longer scored above the cut-off score on the SDQ-20 at post-treatment, i.e., they lost their positive screen for a dissociative disorder. These findings coincide with findings in other studies where specific somatoform dissociative symptoms (i.e., seizure frequency) [22] or psychological dissociative symptoms, e.g., [8,9], proved to decrease over the course of a trauma-focused treatment. Lynch, Forman, Mendelsohn, and Herman [42] already noted that PTSD symptoms and dissociative symptoms tend to move together during treatment, confirmed by the here observed significant correlation between the decrease in PTSD symptoms and somatoform dissociative symptoms. As suggested before [9], this might indicate that dissociative symptoms should be considered as being associated with PTSD, rather than a distinct form of PTSD.

Furthermore, the results showed that the dissociative subtype of PTSD (i.e., psychological dissociative symptoms) was more often present among those with high levels of somatoform dissociative symptoms than those with low levels of somatoform dissociative symptoms. However, almost 25% of those with a high level of somatoform dissociative symptoms did not meet the criteria of the dissociative subtype of PTSD. This is in line with the notion by Holmes et al. [12] that both phenomena can occur together but also in isolation of each other. Therefore, it is important that studies on dissociation and PTSD also include measures of somatoform dissociation. Future research might shed light on the dynamics between PTSD symptoms, psychological dissociative symptoms, and somatoform dissociative symptoms through symptom-based analyses, for example, a network model approach. Recent studies in this area showed, for instance, that psychological dissociative symptoms cluster together with PTSD symptoms, especially re-experiencing [43,44]. However, these studies did not include somatoform dissociative symptoms, which would be an interesting and important follow-up step.

This is the first study that examines somatoform dissociation in relation to PTSD treatment outcome. Its relevance was shown by the direct clinical implications, namely that, when replicated, PTSD patients with severe somatoform dissociative symptoms do not have to be excluded from evidence-based, trauma-focused treatments. This is important, given that somatoform dissociative symptoms are common among traumatized individuals [45,46]. However, when interpreting the results of the current study, some limitations need to be taken into account. The major limitation is the lack of sufficient follow-up data. Yet, the purpose of the present study was to investigate the extent to which PTSD patients with somatoform dissociative symptoms would respond to trauma-focused treatment in comparison to patients without somatoform dissociative symptoms. Studying only post-treatment outcomes enabled us to analyze moderating effects in a very well controlled treatment period. It is nonetheless very important for future studies to include non-selective follow-up data, in order to also examine the long-term course of somatoform dissociative symptoms. Another limitation was the use of a self-report measure for somatoform dissociation. Although the SDQ is a well-validated measure, and scores above the cut-off score of the SDQ proved indicative of a dissociative disorder, the SDQ is a screening measure and not an instrument for classifying dissociative disorders. Therefore, our results cannot be generalized to patients with a dissociative disorder. A third limitation was the lack of randomization in this study. Future research should compare treatment programs such as the one used in this study with a phase-based approach in the form of a randomized clinical trial, in order to be able to draw conclusions regarding possible superiority in treatment approach. This was not the aim of this study, and our study design was therefore different. Nonetheless, our results still contribute significantly, since we found no difference in PTSD symptom decrease after an intensive trauma-focused treatment between those with somatoform dissociative symptoms and those without. Another limitation is that our findings were found in the context of an intensive treatment program, so it is unclear whether our results can be generalized to trauma-focused treatments that are delivered in a typical outpatient format with weekly sessions. One strength of the present study is first and foremost that this is the first study to include somatoform dissociation in the examination of predictors for PTSD treatment outcomes. Additional strengths include the large sample size, the diversity of the sample regarding trauma history, and the use of robust and valid measuring instruments. What is more, in addition to pre- and post-treatment measures, changes in somatoform dissociation symptom severity were also measured during the treatment program.

## 5. Conclusions

In conclusion, the present study showed no difference in PTSD symptom decrease during and after an intensive trauma-focused therapy between those with a high level versus a low level of somatoform dissociation, and the severity of somatoform dissociative symptoms was not predictive of treatment outcome. Moreover, trauma-focused treatment also led to a significant decrease in somatoform dissociative symptoms. These results suggest that patients with PTSD and severe somatoform dissociative symptoms should not be excluded from trauma-focused therapy.

## Figures and Tables

**Table 1:** Baseline characteristics of the ITT sample used for main analysis.

	Total Sample (*n* = 219)	Positive Screen on SDQ-20 (*n* = 29)	Negative Screen on SDQ-20 (*n* = 190)
Gender Women (%)	174 (79.5%)	25 (86.2%)	149 (78.4%)
Mean age (SD)	39.73 (12.27)	36.34 (11.51)	40.25 (12.32)
Three most frequent types of trauma (%)			
Physical abuse	90%	96.6%	88.9%
Sexual abuse	82.2%	100%	79.5%
Armed violence	55.3%	62.1%	54.2%
Comorbidity			
Depression	53.0%	62.1%	51.6%
Anxiety disorder	48.4%	31.0%	51.1%
Suicidality	74.4%	89.7%	72.1%
Dissociative subtype (%)	101 (46.1%)	22 (75.9%)	79 (41.6%)

Note. ITT = intention-to-treat. Types of trauma were measured using the Life Events Checklist for DSM-5 (LEC). Comorbidity was measured using the Mini-International Neuropsychiatric Interview (MINI). The dissociative subtype was assessed using the Clinician-Administered PTSD Scale (CAPS). For the Somatoform Dissociation Questionnaire-20 (SDQ-20), a cut-off score of 35 was applied.

**Table 2:** Mean scores and SDs on the main outcome measures.

		Pre-Treatment *M* (*SD)*	Post-Treatment *M* (*SD*)	Cohen’s *d*
CAPS (*SD*)	Positive screen	45.10 (7.16)	18.07 (13.30)	2.53
	Negative screen	42.94 (7.49)	15.62 (14.88)	2.32
	Total	43.22 (7.47)	15.94 (14.68)	2.34
SDQ-20 (*SD*)	Positive screen	40.93 (6.50)	27.38 (6.82)	2.03
	Negative screen	25.39 (4.13)	22.59 (3.56)	0.79
	Total	27.45 (6.93)	23.22 (4.43)	0.73

## Data Availability

The data presented in this study are available on request from the corresponding author. The data are not publicly available due to lack of permission from participants to share the data publicly.
